# Application of nonsense-mediated primer exclusion (NOPE) for preparation of unique molecular barcoded libraries

**DOI:** 10.1186/s12864-017-3815-2

**Published:** 2017-06-05

**Authors:** Dmitriy A. Shagin, Maria A. Turchaninova, Irina A. Shagina, Mikhail Shugay, Andrew R. Zaretsky, Olga I. Zueva, Dmitriy A. Bolotin, Sergey Lukyanov, Dmitriy M. Chudakov

**Affiliations:** 1 Pirogov Russian National Research Medical University, Moscow, Russia; 2 Shemiakin-Ovchinnikov Institute of Bioorganic Chemistry, Russian Academy of Science, Moscow, Russia; 3 Evrogen JSC, Moscow, Russia; 4 Central European Institute of Technology, Masaryk University, Brno, Czech Republic; 5 Skolkovo Institute of Science and Technology, Moscow, Russia

**Keywords:** High-throughput sequencing, Unique molecular identifiers, Targeted resequencing, PCR

## Abstract

**Background:**

Recently we proposed efficient method to exclude undesirable primers at any stage of amplification reaction, here termed NOPE (NOnsense-mediated Primer Exclusion). According to this method, added oligonucleotide overlapping with the 3′-end of unwanted amplification primer (NOPE oligo) simultaneously provides a template for its elongation. This elongation disrupts specificity of unwanted primer, preventing its further participation in PCR. The suggested approach allows to rationally manage the course of PCR reactions in order to facilitate analysis of complex DNA mixtures as well as to perform multistage PCR bypassing intermediate purification steps.

**Results:**

Here we apply NOPE method to DNA library preparation for the high-throughput sequencing (HTS) with the PCR-based introduction of unique molecular identifiers (UMI). We show that NOPE oligo efficiently neutralizes UMI-containing oligonucleotides after introduction of UMI into sample DNA molecules, thus allowing to proceed with further amplification steps without purification and associated loss of starting material. At the same time, NOPE oligo does not affect the efficiency of target PCR amplification.

**Conclusion:**

We describe a simple, robust and cheap modification of UMI-labeled HTS libraries preparation procedure, that allows to bypass purification step and thus to preserve starting material which may be limited, e.g. circulating tumor DNA, circulating fetal DNA, or small amounts of isolated cells of interest. Furthermore, demonstrated simplicity and robustness of NOPE method should make it popular in various PCR protocols.

## Background

Unique molecular identifiers (UMI) have revolutionized high-throughput sequencing (HTS) strategies. In UMI approach [1–3], each DNA or RNA molecule of a sample gets a unique label at the very first step of library preparation. Once encoded by UMIs, the original sample can be amplified, while the ability to track and distinguish the primary templates through library preparation and sequencing is retained.

UMI provides unprecedented enhancement of the downstream HTS data analysis, by allowing to quantify starting molecules, to eliminate quantitative biases caused by PCR and sequencing, to normalize data for comparative analysis, and to eliminate accumulated PCR and sequencing errors. UMI approach is particularly important in reliable detection of rare gene variants in complex libraries. It can be extremely useful in various targeted resequencing applications, such as cancer [4] and prenatal [5] diagnostics, analysis of heterogeneity and variability of tumors [6], bacterial [7] and viral [8] infections, microbiomes [9], and evolutionary studies [10].

Although UMI is a very promising tool for dealing with HTS libraries for targeted resequencing, there are some technical issues that may complicate their use in practical applications. In particular, it is critical that each UMI must be incorporated into DNA or cDNA template molecule only at the very first stage of reaction, in order to uniquely label the true starting molecule. It means that UMI-containing primers or ligated adaptors should only be active in the first cycle of PCR, or during cDNA synthesis, or during adaptors ligation. Participation of UMI-containing oligonucleotides in subsequent PCR cycles in the course of library preparation would create artificial “diversity” of starting molecules, undermining the following analysis algorithms. Thus application of UMI in HTS library preparation procedure necessarily requires complete removal of UMI-containing oligonucleotides from the reaction before proceeding to the PCR amplification.

Several approaches employing selective removal or suppression of unwanted priming oligonucleotides in the course of PCR amplification have been developed. Column-based purification, SPRI (solid phase reversible immobilization)-based purification, or enzymatic digestion (uracil-DNA-glycosylase, exonuclease) which also requires purification step, can be used, but all these methods are associated with potential loss of material.

In those situations when starting material is limited, it is preferable to proceed to the PCR without purification. A wide range of blocked oligonucleotides can be used to inhibit undesired PCR amplification without purification stage. Those can be oligonucleotides that are modified so that they do not serve as an initiation point for polymerization of a complementary strand by Taq polymerase or similar enzymes. A number of modifications were suggested to inactivate oligonucleotides, including 3′-phosphate group [11], and chemically reversed 3′-terminal nucleotide (3′ to 5′)/inverted end [12] peptide nucleic acids (PNAs), locked nucleic acids (LNAs) etc. Such modified oligonucleotides may compete with the undesirable amplification primers for annealing site or prevent elongation by binding onto the target fragment between the two amplification primers [13–19]. However, these techniques can be implemented only for suppression of external primers before proceeding to internal primers during nested PCR. In the case of step-out PCR, as it is required when dealing with UMI, such blocking oligonucleotides would interfere with subsequent amplification process.

**Fig. 1:**
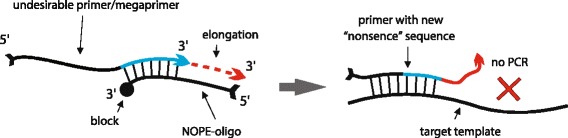
General principle of NOPE method. NOPE oligo is complementary to the 3′ end of the unwanted primer. Upon binding to the 3′ end of the primer, NOPE oligo provides a template for the primer extension, thereby producing new “nonsense” 3′-end (i.e. the 3′-end that is non-complementary to the amplified template)

Recently we reported the method for selective, efficient, purification-free neutralization of undesirable primers in PCR reactions [20], here called NOPE (NOnsense-mediated Primer Exclusion, Fig. 1). NOPE approach was successfully applied by another research group for the same application as in our primary work - suppression of mega-primers in alpha-beta TCR pairs matching by overlap-extension [21]. Furthermore, NOPE can be also used for a wide variety of applications where it is needed to block undesirable primers or mega-primers. In particular, it can be employed in multistage experiments for blocking the priming activity of DNA molecules remaining from previous reaction stage, e.g. for nested PCR, step-out PCR, PCR after adaptors ligation, etc.

Here we applied NOPE for neutralization of UMI-containing primer remaining after its PCR-based incorporation into template DNA molecules. We found that addition of NOPE oligo allows to achieve nearly complete elimination of remaining UMI-containing primers without cleanup step. All alternative techniques require more handling or have a higher cost compared to NOPE. In those cases where purification of reaction mixture is impossible/highly undesirable due to the rarity of samples, NOPE becomes an indispensable tool.

## Results

**Fig. 2:**
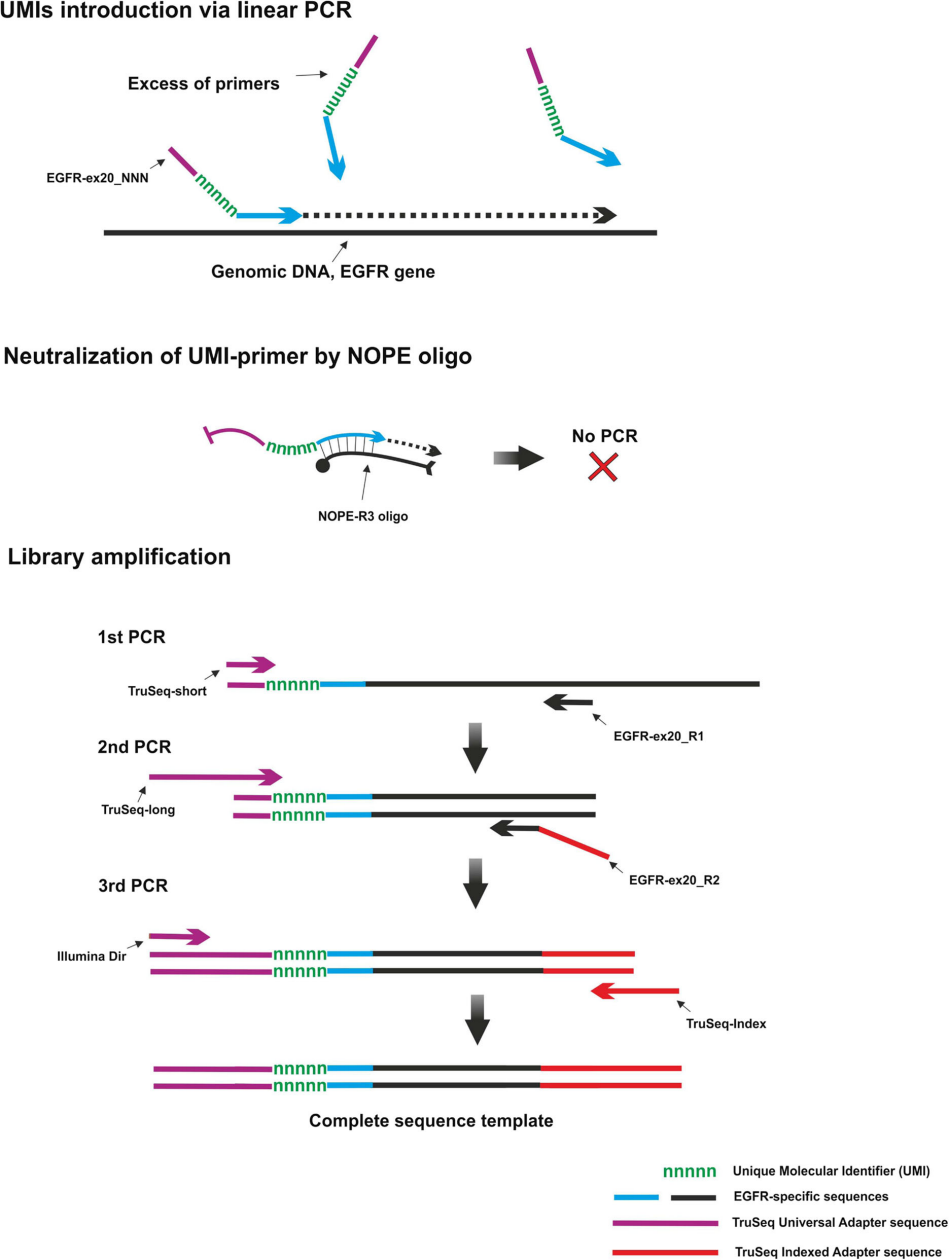
Exemplary experiment. DNA library construction and neutralization of UMI after PCR-based UMI introduction. Genomic DNA samples are amplified using 10 cycles of linear PCR with EGFR exon 20 specific primer modified to include UMI and partial Illumina sequencing adapter. Remaining UMI-primer is neutralized using 10 cycles of annealing/elongation in the presence of NOPE oligo. Next, 3 rounds of PCR are performed for amplification of EGFR gene fragments and introduction of complete Illumina adapters and indexes sequences

**Fig. 3:**
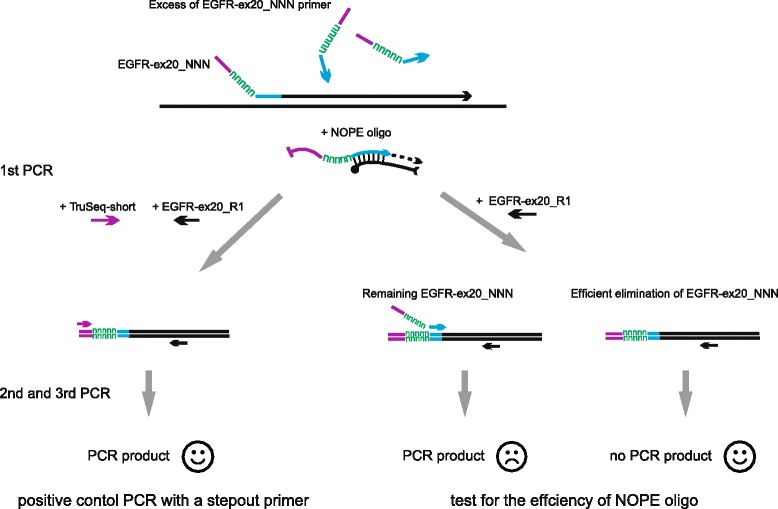
Scheme of NOPE oligos comparative testing

To demonstrate the reliability of NOPE in targeted resequencing, we designed an exemplary experiment, where NOPE oligo was used for elimination of UMI-containing primer after introduction of UMI into the template DNA via linear PCR, and before further amplification (Fig. 2). This experimental system was further employed in our current project on identification of tumor mutations with targeted resequencing of EGFR (epidermal growth factor receptor) gene. Mutations in tyrosine kinase domain of EGFR gene (18–22 exons) are important predictive markers for clinical benefit from EGFR tyrosine kinase inhibitor (TKI) therapies in development for non-small cell lung cancer (NSCLC), one of the most common and deadly cancer [22]. Peripheral blood mononuclear cell (PBMC) DNA of a healthy donor was used in model experiments for demonstration of NOPE oligo efficiency. UMI-barcoding of analyzed DNA was performed using linear PCR followed by amplification of exon 20 human EGFR gene. In this design, efficient neutralization of the UMI-containing primer EGFR-ex20_NNN by a NOPE oligo should prevent further amplification of the target template with a pair of EGFR-ex20_NNN and EGFR-ex20_R1 primers, while amplification using a step-out TruSeq-short primer and EGFR-ex20_R1 primer should remain efficient (Fig. 3). Oligonucleotides used in our study are listed in Table 1.

**Table 1:** Oligonucleotides

Primer	Application	Sequence
Primers for linear PCR		
EGFR-ex20_NNN	PCR-based introduction of UMI (UMI-containing primer), contains partial sequence of TruSeq Illumina adapter	ACACGACGCTCTTCCGATCTNNNNNNNNNNNNNNCATCTGCCTCACCTCCACCGT
EGFR- ex18-1reg_NNN		ACACGACGCTCTTCCGATCTNNNNNNNNNNNNNNGAAGCTCCCAACCAAGCTC
EGFR- ex19-1reg_NNN		ACACGACGCTCTTCCGATCTNNNNNNNNNNNNNNCTGTCATAGGGACTCTGGAT
EGFR -ex21_R_NNN		ACACGACGCTCTTCCGATCTNNNNNNNNNNNNNNTCTTTCTCTTCCGCACCCAG
EGFR -ex22_NNN		ACACGACGCTCTTCCGATCTNNNNNNNNNNNNNNCATCCCAAGGTGCCTATCAA
NOPE oligos		
NOPE-R1	Neutralization of EGFR-ex20_NNN	GCACGCGTCGACGGTGGAGGTG-BHQ1
NOPE-R2		ACACGACGCTCTTCCGATCTACGGTGGAGGTGAGGCAG-BHQ1
NOPE-R3		GCACGCGTCGACGGTGGAGGTGAGGCAG-BHQ1
NOPE-R0		ACGGTGGAGGTGAGGCAG-BHQ1
NOPE-EGFR- ex18	Neutralization of EGFR-ex18-1reg_NNN	TCACACGTCTGAGCTTGGTTGGGAGCTTC-BHQ1
NOPE-EGFR-ex19	Neutralization of EGFR- ex19-1reg_NNN	ATACTCTTCGATCCAGAGTCCCTATGACAG-BHQ1
NOPE- EGFR-ex21	Neutralization of EGFR-ex21_R_NNN	CTTGACGTCACTGGGTGCGGAAGAGAAAGA-BHQ1
NOPE- EGFR-ex22	Neutralization of EGFR-ex22_NNN	ACTCTCATCCTTGATAGGCACCTTGGGATG-BHQ1
Primers for the 1st PCR		
EGFR-ex20_R1	Reverse nested EGFR-ex20-specific primer 1	TGTTCCCGGACATAGTCCAG
EGFR-ex18-1reg_ R1	Reverse nested EGFR-ex18-specific primer 1	AACGCACCGGAGCCCAGCACT
EGFR-ex19-1reg_R1	Reverse nested EGFR-ex19-specific primer 1	GATTTCCTTGTTGGCTTTCGGA
EGFR-ex21_F1	Forward nested EGFR-ex21-specific primer 1	CTGGTGAAAACACCGCAGCA
EGFR-ex22_R1	Reverse nested EGFR-ex22-specific primer 1	GCTCCAGACATCACTCTGGT
TruSeq-short	Step-out primer 1	TACACGACGCTCTTCCGATCT
Primers for 2nd PCR		
EGFR-ex20_R2	Reverse nested EGFR-ex20-specific primer 2, contains partial sequence of TruSeq Illumina adapter	GTGACTGGAGTTCAGACGTGTGCTCTTCCGATCTACATAGTCCAGGAGGCAGC
EGFR-ex18-1reg_R2 Br	Reverse nested EGFR-ex18-specific primer 2, contains partial sequence of TruSeq Illumina adapter	GTGACTGGAGTTCAGACGTGTGCTCTTCCGATCTCGGAGCCCAGCACTTTGATC
EGFR-ex19-1reg_R2 Br	Reverse nested EGFR-ex19-specific primer 2, contains partial sequence of TruSeq Illumina adapter	GTGACTGGAGTTCAGACGTGTGCTCTTCCGATCTTGTTGGCTTTCGGAGATGTT
EGFR-ex21_F2 Br	Forward nested EGFR-ex21-specific primer 2, contains partial sequence of TruSeq Illumina adapter	GTGACTGGAGTTCAGACGTGTGCTCTTCCGATCTAAACACCGCAGCATGTCAAG
EGFR-ex22_R2 Br	Reverse nested EGFR-ex22-specific primer 2, contains partial sequence of TruSeq Illumina adapter	GTGACTGGAGTTCAGACGTGTGCTCTTCCGATCTACATCACTCTGGTGGGTATAG
TruSeq-long	Step-out primer 2, contains sequence of TruSeq Illumina adapter	AATGATACGGCGACCACCGAGATCTACACTCTTTCCCTACACGACGCTCTTCCGATCT
Primers for 3rd PCR (introducing Illumina indexes)		
TruSeq-Index1	Reverse step-out primer, anneals on the EGFR-ex20_R2, contains complete sequence of TruSeq Illumina adapter with sample index	CAAGCAGAAGACGGCATACGAGATCGTGATGTGACTGGAGTTCAGACGTGT
Illumina-Dir	Forward step-out primer 3	AATGATACGGCGACCACCGAGATC
Real time PCR probes		
Z-IGFR ex20	Real time PCR probe for exon 20.	Fam-AGCTCATCACGCAGCTCATGCCCTT-BHQ1
Z-TruUni	Universal real time PCR probe for TuSeq adapter	Fam-ACACTCTTTCCCTACACGACGCTCTT-BHQ1

### Design of NOPE oligo

NOPE oligo should be modified at its 3′-end, resulting in full inhibition of its enzymatic elongation. Modification must be stable and resistant to degradation or enzymatic removing after synthesis. Earlier we tried several variants of the 3′ end modification (Ref. [20] and unpublished data) and finally opted for black hole quencher 1 (BHQ1), which prevents elongation without noticeably influencing the oligos annealing properties.

NOPE oligo may overlap completely with the undesired primer or only partially with its 3′-end. The length and content of a “nonsense” template (5′-end of a NOPE oligo that serves for the elongation and neutralization of the unwanted primer) may also vary.

**Fig. 4:**
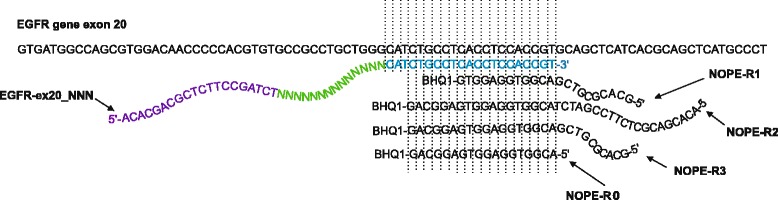
EGFR gene region of interest. Region of human EGFR gene sequence showing the location of the UMI-containing primer EGFR-ex20_NNN and 4 different NOPE oligos

To get better understanding of how efficiency of a NOPE oligo depends on its structure, we tested different possibilities for 3′-overlap and 5′-extension. We designed four different NOPE oligos for neutralization of UMI-containing EGFR-ex20_NNN primer: NOPE-R1, NOPE-R2, NOPE-R3, and NOPE-R0. The latter oligo completely lacked the part for the “nonsense” elongation of the EGFR-ex20_NNN primer. Region of human EGFR gene sequence showing the location of UMI-containing primer and four different NOPE oligos is presented on the Fig. 4.

EGFR gene fragment was amplified as described on Fig. 2. EGFR-ex20_NNN primer neutralization step was performed using one of the NOPE oligos. To detect the minimal NOPE oligos concentration required for efficient elimination of the EGFR-ex20_NNN primer present at concentration 0.1 μM, we carried out a set of neutralisation PCRs with NOPE oligos concentrations ranging from 0 to 1.5 μM. After neutralization step, all reactions were divided into two halves (Fig. 3). One half was subsequently amplified (1st PCR) using step-out TruSeq-short primer and reverse primer EGFR-ex20_R1 and showed the overall efficiency of amplification (control). The other half was amplified using only one reverse EGFR-ex20_R1 primer, and reflected the completeness of EGFR-ex20_NNN elimination by a NOPE oligo (test).

**Fig. 5:**
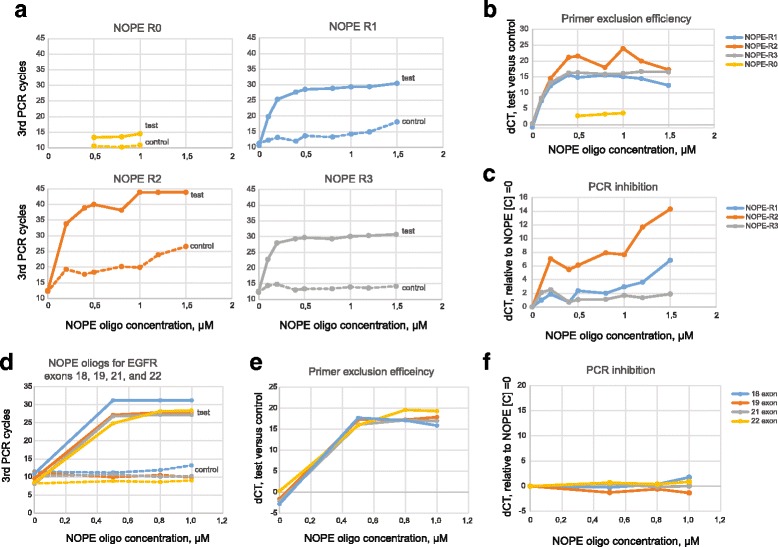
Real-time PCR analysis of NOPE oligos efficiency. Results of the 3rd PCR are shown. Typical results are shown for the two independent experiments each performed in two replicas. See Table 1 for all oligonucleotides used. **a** Four different NOPE oligos (NOPE-R0, R1, R2, R3) performance in amplification of EGFR gene exon 20 fragment. Dotted lines represent positive control reaction containing NOPE oligo and two amplification primers: forward TruSeq-short and reverse EGFR-ex20-R1 in the 1st PCR. Solid lines represent test reactions containing NOPE oligo and only reverse primer EGFR-ex20_R1 in the 1st PCR. **b** Comparison of EGFR-NNN-ex20 primer exclusion efficiency by the four NOPE oligos. dCt between control reaction with two amplification primers in the 1st PCR and test reaction with only reverse primer in the 1st PCR reflects efficiency of EGFR-NNN-ex20 primer exclusion. **c** Comparison of the NOPE oligos inhibitory effect on amplification of target EGFR gene exon 20 fragment. dCt between amplification with two EGFR exon 20 amplification primers with and without NOPE oligo reflects inhibition of the target DNA amplification by NOPE oligo. **d** NOPE oligos performance in amplification of EGFR gene exons 18, 19, 21, and 22 fragments. Dotted lines represent control reactions containing NOPE oligo and two amplification primers in the 1st PCR: TruSeq-short and complementary EGFR primer. Solid lines represent test reactions containing NOPE oligo and a single EGFR primer in the 1st PCR. **e** EGFR-NNN primers exclusion efficiency by NOPE oligos for EGFR gene exons 18, 19, 21, and 22 fragments. dCt between control reaction with two amplification primers in the 1st PCR and test reaction with only the reverse primer in 1st PCR reflects efficiency of of EGFR-NNN primer exclusion. **f** NOPE oligos inhibitory effect on amplification of target fragments of EGFR gene exons 18, 19, 21, and 22. dCt between reactions containing NOPE oligo and two EGFR amplification primers and reactions containing only EGFR amplification primers reflects inhibition of the target DNA amplification by NOPE oligo

**Fig. 6:**
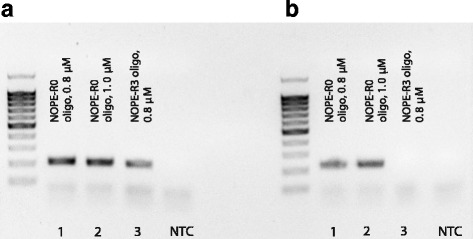
Comparison of NOPE-R3 and NOPE-R0 oligo performance. Lanes A1-A3 represent reactions containing two primers in the 1st PCR – forward TruSeq-short and reverse EGFR-ex20-R1; lanes B1-B3 represent reactions containing only the reverse primer EGFR-ex20-R1. NTC – non template control. See Methods for details. Note absence of PCR product in B3 reaction that has been amplified for 11 more cycles compared to A3 and 9 more cycles compared to B1

Real-time PCR analysis performed with EGFR exon 20 specific TaqMan probe showed that primer exclusion by NOPE-R0 oligo which completely lacks the 5′-nonsense sequence was inefficient (Figs. 5a,b and 6). The difference in the number of amplification cycles for the PCR with and without TruSeq-short primer was only about 2–3 cycles. This demonstrated that effect of neutralisation based solely on the competition for the EGFR-ex20_NNN annealing between the template and blocking oligo is minimal.

At the same time, all three blocking oligos carrying the 5′-extension efficiently excluded EGFR-ex20_NNN primer from reaction (Fig. 5a,b), demonstrating robustness of the NOPE approach (see Fig. 5a,b). In the presence of TruSeq-short primer, inhibition of the target PCR amplification by NOPE-R1 and NOPE-R3 oligos was negligible, demonstrating that these oligos do not influence the efficiency of target PCR reaction (Fig. 5a, control, Fig. 5c, Fig. 6a). At the same time, NOPE-R2 notably inhibited target PCR, presumably interfering with the reaction in a sequence-specific manner. This indicates that newly designed NOPE oligo should be tested for side effects, as well as any new primer.

Simultaneously with the different NOPE oligos comparison we have determined their effective concentrations. The concentration of NOPE oligo should be sufficient to elongate nearly all molecules of the undesirable primer. At the same time, too high concentrations of the NOPE oligo could have an inhibiting effect on further amplification of target DNA. Increase of the NOPE oligos concentration from 0.1 μM to 0.5 μM consistently improved the efficiency of EGFR-ex20_NNN primer neutralisation (Fig. 5), while further increase of NOPE oligo concentration yielded poor returns.

To further verify universality of NOPE approach, we have additionally designed and tested NOPE-oligos for fragments of EGFR exons 18, 19, 21, and 22. All NOPE oligos demonstrated efficient exclusion of UMI-introducing primer at concentration of 0.5–1 μM (Fig. 5d,e), with minimal inhibition of target PCR (Fig. 5f), thus confirming the general robustness of NOPE method.

### EGFR gene fragments library analysis by HTS

Designed pipeline was applied to the EGFR-ex20 gene fragment library preparation procedure for the four circulating cell-free DNA samples of patients with lung cancer together with the control for assessment of NOPE-R3 efficiency. NOPE-R3 oligowas used at concentration 0.8 μM for suppression of 0.1 μM EGFR-ex20_NNN primer. Bands of anticipated size were observed for all samples. No PCR product was detected in control lane (without TruSeq-short primer in the 1st PCR). The final difference between the number of amplification cycles after three rounds of amplification for target samples and the control was 20–22. This internal control confirmed that addition of NOPE oligo removes the UMI-containing primer from the reaction very effectively, if not completely. For comparison, we prepared the same EGFR-ex20 libraries using alternative method of EGFR-ex20_NNN primer elimination with *E. coli* Exonuclease I treatment followed by column purification (according to Ref. [3], with minor modifications, details on the method to be published elsewhere). 20 ng of circulating cell-free DNA was used per each sample. 1/5 of the linear PCR product was used in case of NOPE. 1/2 of the column-purified linear PCR product was used in case of ExoI treatment. The resulting libraries of EGFR gene fragment were analyzed using paired-end Hiseq2500 Illumina sequencing.

**Fig. 7:**
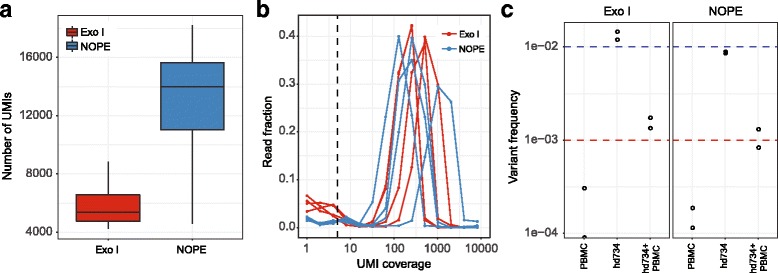
HTS data analysis. EGFR-ex20 gene libraries were prepared in parallel using NOPE and ExoI approaches for EGFR-ex20_NNN primer exclusion. **a** The number of detected UMI sequences with a sufficient (5+ read) coverage. **b** UMI coverage distribution showing the fraction of reads (Y axis) tagged with UMIs covered X times. Dashed line shows reads-per-UMI threshold selected for data analysis. **c** Identification of T790 M mutation in EGFR gene in control PBMC DNA, the reference standard and their 9:1 mixture. *Red* and *blue* lines show the expected mutation frequency in the mixture and pure reference standard sample, respectively

Data analysis was performed using our MAGERI software designed to analyze UMI-tagged targeted genome re-sequencing data [23]. In this back-to-back comparison starting with identical amounts of DNA, NOPE method outperformed ExoI method in respect of the number of successfully labeled and amplified molecules (Fig. 7a), even in spite of the lower amount of the linear PCR product used in the 1st PCR. This probably results from damage of template molecules by ExoI and material loss during purification step used after ExoI treatment.

At the same time, narrow shape of the UMI coverage peaks (Fig. 7b) confirms absence of notable inclusion of EGFR-ex20_NNN primer in PCR amplification after initial incorporation during linear PCR.

Next, we prepared EGFR-ex20 gene fragment libraries from the healthy PBMC DNA sample, standard reference genomic DNA (Tru-Q 7 1% Tier reference mutation panel, Horizon Dx, USA; Cat. ID HD734), and the same reference DNA mixed with PBMC DNA in 1:9 proportion. Libraries were prepared by ExoI method and NOPE method, sequenced on Illumina HiSeq2500 and analyzed using MAGERI software. Both methods successfully identified mutation EGFR_T790 M present at frequency 0.01 in Tru-Q 7 1% Tier reference mutation panel and at 0.001 frequency in reference panel mixed 1:9 with a healthy donor PBMC (Fig. 7c). These results demonstrate applicability and efficiency of NOPE method for targeted re-sequencing with UMI.

## Discussion

Today UMI approach is widely used in multiple applications, including genome, transcriptome [2, 3, 24] and microbiome [9] analysis, immune repertoires sequencing [25–32], detection of low-frequency mutations [3, 33], studying the incidence of transcription [34], primer synthesis [3], or sequencing [35] errors.

PCR-based introduction of UMI may use target-specific primers modified to include UMI and Illumina sequencing adaptors. The resulting PCR product is a sequencing-ready library that does not require additional adaptors ligation step thus reducing the overall cost, time and complexity of the experiment. Here we focused on further rationalization of UMI-labeled library preparation for targeted resequencing and described application of NOPE method for processing of DNA library with PCR-based UMI introduction.

We showed that addition of NOPE oligo efficiently resolves the problem of UMI-containing primers remaining after PCR-based introduction of molecular identifiers. We implemented this technology in our current HTS project related to the tumor mutations profiling. On the exemplary system we described library preparation procedure for targeted resequencing and cancer mutation detection in single EGFR gene. However, the same method could be easily adapted for multiple genes library preparation.

Advantages of the proposed method become critical when it comes to the low-copy number DNA samples. Thus from a practical perspective, NOPE oligos can be widely applied in all those cases where purification of reaction may result in material loss. In particular, NOPE method should be efficient for molecular assessment of circulating cell-free tumor or fetal DNA, or minor cell samples of interest.

Furthermore, NOPE method offers avenues in various fields beyond HTS. For example, this approach could be used for nested PCR free of interim dilution and purification steps. Inhibition of the 1st PCR primers by NOPE oligos added right to the 1st PCR mixture allows to proceed with the 2nd PCR in the same tube just by adding of nested primers. Another applicational example is inhibition of megaprimers in those particular cases where mega-priming process may adversely affect subsequent reactions [20, 21]. Other applications may be invented. We believe that simplicity and robustness of the NOPE method should make it popular in various PCR protocols.

## Conclusions

In the present study, we implemented our NOPE method for the UMI-based library preparation for HTS. Our results show that NOPE approach allows to achieve nearly complete elimination of remaining UMI-containing primers from the reaction without cleanup step and thus testify the utility of NOPE oligos in HTS analyses, especially where low-copy number DNA samples may compromise the results.

## Methods

### UMI-barcoded library preparation using NOPE oligo

*Specimens collection and DNA extraction.* Samples of healthy donors PMBC DNA, circulating cell-free DNA, and reference genomic DNA (Tru-Q 7 (1.3% Tier) Reference Standard, Horizon) were used in experiments. Circulating cell-free DNA was isolated from the blood plasma of patients with adenocarcinoma at Molecular Biology & Cytogenetics Lab, Russian Center for Roentgenology & Radiology (Moscow, Russian Federation). DNA extraction was performed with the use of QIAamp Circulating Nucleic Acid Kit (Qiagen).

*Introduction of UMI* via *linear PCR.* 25 ng of gDNA was used for the reaction. Besides DNA, linear PCR (50 μL) contained also: 1× Tersus Buffer (Evrogen), 200 μM of each dNTP, 5 U of Tersus polymerase (Evrogen) and 0.1 μM of the forward primer EGFR-ex20_NNN. Thermal cycling conditions were: 1 min at 95 °C; 10 cycles of 15 s at 95 °C, 20 s at 60 °C, 30 s at 72 °C; and finally 2 min at 72 °C. Nontemplate control was included.

*Neutralization of remaining EGFR-ex20_NNN and NOPE oligo concentration optimization.* For neutralization of remaining UMI-containing primers after introduction of UMI into EGFR gene fragments, 0.1–1.5 μM of four different NOPE oligos were added to the reactions. NOPE oligos concentrations and NOPE oligo/EGFR-ex20_NNN primer ratios used in the experiment are shown in Table 2. The temperature settings were: 10 cycles of 30 s at 60 °C, 20 s at 65 °C.

**Table 2:** NOPE oligo quantities tested during concentration optimization

№ of sample	1	2	3	4	5	6	7	8
NOPE-R3/EGFR-ex20_NNN ratio	1:1	2:1	4:1	5:1	8:1	10:1	12:1	15:1
NOPE-R3 oligo concentration, μM	0.1	0.2	0.4	0.5	0.8	1.0	1.2	1.5

### Library amplification

1st PCR. After neutralization of UMI-containing primer, reactions were divided into two halves. For further libraries amplification, 0.2 μM of the forward primer TruSeq-short and 0.2 μM of the reverse primer EGFR-ex20_R1 were added to the positive control half of the reaction. Only reverse primer EGFR-ex20_R1 was added to the test half of the reaction (Fig. 3). The following cycle settings were used: 30 cycles of 15 s at 95 °C, 20 s at 62 °C and 30 s at 72 °C, final extension for 2 min at 72 °C.

2nd PCR. The second amplification was carried out using 1 μL of unpurified 1000-fold diluted product of the 1st PCR. 25 μL reaction contained: 1× Tersus Buffer, 200 μM of each dNTP, 5 U of Tersus polymerase, 0.2 μM of the forward primer TruSeq-long and 0.2 μM of the reverse primer EGFR-ex20_R2. The following cycle settings were used: denaturation, 1 min at 95 °C, followed by 11 cycles of 15 s at 95 °C, 20 s at 63 °C and 30 s at 72 °C. Final extension was 2 min at 72 °C.

3rd PCR. During the third amplification Illumina indexes were incorporated into DNA library. 3rd amplification was carried out using 2 μL of unpurified 1000-fold diluted product of the 2nd PCR. 25 μL reaction contained: 1× qPCRmix-HS (Evrogen), 0.2 μM of the Illumina-Dir primer, 0.2 μM of the TruSeq-Index 1 primer and 0.2 μM of the TaqMan probe. Real-time PCR was performed using ABI PRISM 7500 Sequence Detection System (Applied Biosystems). Reactions were set up in duplicate in two independent experiments. The following cycle settings were used: 3 min of polymerase activation at 95 °C, followed by 45 cycles of 15 s at 95 °C, 30 s at 63 °C and 1 min at 72 °C. Fluorescence signal was collected at every annealing step.

Testing of NOPE oligos for EGFR exons 18, 19, 21, 22 was performed under the same reaction conditions that have been described above for EGFR exon 20.

#### Comparison of NOPE-R3 and NOPE-R0 oligo performance (Fig. 6)

Non-template control was included. After UMI-containing primer neutralization step, all reactions were divided into two halves. To the one half of reaction, 0.1 μM of the forward primer TruSeq-short and 0.1 μM of the reverse primer EGFR-ex20_R1 were added in the 1st PCR mix. To the second half, only 0.1 μM of the reverse primer EGFR-ex20_R1 was added in 1st PCR mix. 30 amplification cycles were performed for all samples during 1st PCR. The following cycles number were used during 2nd PCR: for A1, A2, A3, B1, B2–11; for B3–20. The following number of PCR cycles was used during the 3rd PCR: for A1, A2, A3–11; for B1, B2, B3–13. The resulting PCR product was visualized on agarose gel.

## Abbreviations

BHQ1: Black hole quencher 1
EGFR: Epidermal growth factor receptor
HTS: High-throughput sequencing
LNA: Locked nucleic acids
NOPE: Nonsense-mediated primer exclusion - method reported here
NSCLC: Non-small cell lung cancer
PBMC: Peripheral blood mononuclear cells
PCR: Polymerase chain reaction
PNA: Peptide nucleic acids
SPRI: Solid phase reversible immobilization
UMI: Unique molecular identifiers
